# Roux-en-Y gastric bypass alters intestinal glucose transport in the obese Zucker rat

**DOI:** 10.3389/fendo.2022.901984

**Published:** 2022-08-11

**Authors:** Qinghe Meng, Derek M. Culnan, Tamer Ahmed, Mingjie Sun, Robert N. Cooney

**Affiliations:** ^1^ Department of Surgery, State University of New York (SUNY), Upstate Medical University, Syracuse, NY, United States; ^2^ Burn and Reconstructive Centers of America, Jackson, MS, United States; ^3^ Department of Surgery, Pennsylvania State University College of Medicine, Hershey, PA, United States

**Keywords:** RYGB, GLP-1, SGLT1, GLUT2, TAS1R1, TAS1R3, α-gustducin 2

## Abstract

**Introduction:**

The gastrointestinal tract plays a major role in regulating glucose homeostasis and gut endocrine function. The current study examines the effects of Roux-en-Y gastric bypass (RYGB) on intestinal GLP-1, glucose transporter expression and function in the obese Zucker rat (ZR).

**Methods:**

Two groups of ZRs were studied: RYGB and sham surgery pair-fed (PF) fed rats. Body weight and food intake were measured daily. On post-operative day (POD) 21, an oral glucose test (OGT) was performed, basal and 30-minute plasma, portal venous glucose and glucagon-like peptide-1 (GLP-1) levels were measured. In separate ZRs, the biliopancreatic, Roux limb (Roux) and common channel (CC) intestinal segments were harvested on POD 21.

**Results:**

Body weight was decreased in the RYGB group. Basal and 30-minute OGT plasma and portal glucose levels were decreased after RYGB. Basal plasma GLP-1 levels were similar, while a 4.5-fold increase in GLP-1 level was observed in 30-minute after RYGB (vs. PF). The increase in basal and 30-minute portal venous GLP-1 levels after RYGB were accompanied by increased mRNA expressions of proglucagon and PC 1/3, GPR119 protein in the Roux and CC segments. mRNA and protein levels of FFAR2/3 were increased in Roux segment. RYGB decreased brush border glucose transport, transporter proteins (SGLT1 and GLUT2) and mRNA levels of Tas1R1/Tas1R3 and α-gustducin in the Roux and CC segments.

**Conclusions:**

Reductions in intestinal glucose transport and enhanced post-prandial GLP-1 release were associated with increases in GRP119 and FFAR2/3 after RYGB in the ZR model. Post-RYGB reductions in the regulation of intestinal glucose transport and L cell receptors regulating GLP-1 secretion represent potential mechanisms for improved glycemic control.

## Introduction

The Roux-en-Y gastric bypass (RYGB) is a commonly performed bariatric procedure. Many patients demonstrate improvements in type 2 diabetes mellitus (T2DM) shortly after RYGB and prior to significant weight loss (1–3). Early post-RYGB improvements in glycemic control appear to be due in part to enhanced glucagon-like peptide (GLP-1) secretion and improvements in insulin sensitivity (4). Gastrointestinal procedures like ileal interposition are associated with improvements in glycemic control, enhanced GLP-1 secretion, and decreased insulin resistance despite minimal changes in body weight or composition (5, 6). Collectively, these observations raise questions regarding the relative importance of decreased food intake, weight loss, changes in fat depots, and/or adipokine secretion in early post-operative improvements in obesity-related insulin resistance and glycemic control.

Increasing evidence suggests a critical role for the gut in the pathogenesis of T2DM. High fat diets are associated with intestinal inflammation, alterations in barrier function, change in bacterial flora and circulating endotoxemia (7, 8). Gut endocrine function also appears to be altered in obesity and T2DM. While glucose-dependent insulinotropic polypeptide (GIP) levels are not altered in T2DM, function is impaired by reductions in β-cell GIP receptors and post-receptor signaling defects (9, 10). Impaired GLP-1 release and action have also been described in T2DM (11). Increased post-prandial plasma levels of GLP-1 after RYGB are observed in patients and pre-clinical models. Consequently, alterations in incretin synthesis or activity have been posited as important mechanisms for improved insulin sensitivity following RYGB surgery (3, 12).

Increased expression and activity of brush border glucose transporters have also been described in patients and animal models of T2DM (13, 14). However, the effects of RYGB on intestinal glucose transport and transporter expression in experimental models of T2DM have not previously been well characterized. Intestinal glucose transport is regulated by the Na^+^/glucose cotransporters SGLT1 and GLUT2 (14, 15). SGLT1 mediates “active” transport of low concentrations of luminal D-glucose across the brush border membrane (BBM) into the enterocyte (16). The sodium which enters the cell along with glucose is transported across the basolateral membrane into the blood by the Na^+^/K^+^-pump to maintain the driving force for glucose uptake. As glucose accumulates inside the enterocyte, the “passive” transport of glucose into the bloodstream is regulated by the GLUT2 transporter located in the basolateral membrane (15). Some studies suggest apical translocation of GLUT2 following a carbohydrate-rich meal results in mass absorption of luminal glucose across the BBM by GLUT2 (17). Apical translocation of GLUT2 in enterocytes is regulated by luminal sugars, calcium, GLP-2, insulin and sweet taste receptors (18, 19).

The Tas1R family of sweet taste receptors is made up of three G-protein coupled receptors (GPRCs) which are located throughout the gastrointestinal tract on taste buds, enterocytes, and enteroendocrine cells (20–22). The Tas1R1/Tas1R3 heterodimer responds to “sweet tastants” like glucose or artificial sweeteners by signaling through α-gustducin or transducin to activate the phospholipase C or protein kinase pathways (23). Dietary sugars regulate the expression of SGLT1 by a mechanism involving sweet taste receptors and the taste G protein gustducin (24). Intestinal sweet receptors also regulate the apical trafficking of GLUT2 (23). In addition, luminal glucose can also trigger the secretion of incretins like GIP by K cells and GLP-1 by L-cells into the portal venous circulation (25).

The current study examines the effects of RYGB on plasma and portal venous glucose and GLP-1 concentrations, the factors that regulate GLP-1 secretion, intestinal glucose transport and transporters. Our data indicate RYGB-induced reductions in intestinal glucose transport and enhanced post-prandial GLP-1 release contribute to improvements in glucose homeostasis after RYGB in the ZR model. Expression of proglucagon, enzyme proprotein convertase 1/3 (PC 1/3), short chain fatty acids (SCFAs) receptors FFAR2/3 and sweet taste receptor expression were also examined in intestine to understand their role in promoting GLP-1 secretion and in regulating glucose transporter expression after RYGB.

## Materials and methods

### Animal care and surgical procedure

Due to a mutant leptin receptor with impaired satiety, obese Zucker rats (ZRs) have significantly increased food intake compared to lean heterozygous ZR controls. Control animals (sham surgery) were pair-fed (PF) to animals with RYGB surgery because food intake affects nutrient absorption. In this study, two groups (RYGB and sham surgery PF) of obese male ZRs (Charles River Laboratories in Wilmington, MA), 10 to 12 weeks old, were studied. Animals were housed in wire bottom cages to prevent coprophagia. Animals had *ad libitum* access to water and food (Harlan Teklad 2018) except for the pre-test overnight fast and the immediate postoperative period. The animal experiments were approved by the Institutional Animal Care and Use Committee of Pennsylvania State University. The study was performed in accordance with the National Institutes of Health and ARRIVE guidelines on the use of laboratory animals.

Animals were randomly assigned to RYGB or PF groups before surgery. The RYGB procedure was performed as previously described by our laboratory (26). Briefly, rats were fasted overnight the day before surgery. After randomization, rats were weighed and then anesthetized with isoflurane (3% for induction and 1.5% for maintenance). Anesthesia and respiratory status were continuously monitored by a dedicated member of the surgical team to prevent respiratory complications. Ceftriaxone (100 mg/kg, IM, Roche, Nutley, NJ) was administered as a prophylactic antibiotic. Animals were placed on a heating pad during surgery. The abdomen was opened using a midline laparotomy and closed using continuous sutures under sterile conditions. In the sham surgery group, only bowel manipulation was performed followed by abdominal closure. In the RYGB group, the stomach divided to create a reduced (~20%) gastric pouch using a 45 mm GIA stapler (ETS-Flex Ethicon Endo surgery). The small intestine is divided into a biliopancreatic limb (15 cm), an alimentary limb or Roux limb (10 cm), and a common channel (33 cm). Gastrojejunostomy and jejunojejunostomy were performed using interrupted 5.0 silk sutures. 0.5 ml of 0.25% bupivicaine was injected into the surgical incision for analgesia. All rats received warm saline (50 ml/kg, SQ) before the start of surgery, immediately after surgery, and again on *postoperative day 1* (*POD 1*). 0.25% bupivacaine (0.5 ml, IP) was also injected on *POD 1*. Once fully recovered, animals were returned to home cages and housed individually. Body weight was monitored daily from day 0. To facilitate surgical anastomotic healing, animals were initiated on a liquid diet containing Resource and water *ad libitum* approximately 24 hours after surgery, and animals were treated with subcutaneous saline administration. Then, regular chow was started on *POD 3* and food intake was monitored daily. To determine that the effect of RYGB on body weight occurred independently of changes in energy intake, Pair-feeding was employed. The amount of food provided to PF group matched the amount of food consumed by the RYGB rats, and the RYGB group was allowed to eat *ad libitum*. Rats exhibiting excessively reduced food intake, abdominal distention, and/or dehydration early after surgery, they were euthanized and excluded. On *POD 21*, the animals were euthanized under anesthesia with sodium phenobarbital (100 mg/kg, IP). Blood was collected form tail vein by tail snip and from portal vein before (*t_0_*), and 30 min (*t_30_*) after oral gavage with dextrose (1.25 g/kg 25%). Blood were collected using the tubes containing 50 mmol/l EDTA, 12 TIU/ml aprotinin, and 100 µmol/ml dipeptidyl peptidase-4 (DPP-4) inhibitor for the measurements of glucose and GLP-1. Biliopancreatic limb (BP limb), alimentary (Roux limb) and common channel (CC) were collected to keep in liquid nitrogen for protein and RNA analysis or isolate brush-border membrane vesicles (BBMVs) after collection of blood.

### Measurement of GLP-1 and glucose

Total-GLP-1 was measured by multiplexed ELISA (Cat. E: C403P-1, Mesoscale Discoveries) according to the manufacturer’s guidelines. Glucose was measured by Glucose Analyzer (Analox M7, Luenburg, MA)

### Purification of BBMVs

BBMVs were prepared in the same way as described previously (27). Briefly, the intestine was rinsed with ice-cold 300 MHT buffer (300 mM D-mannitol, 10 mM HEPES-Tris, pH 7.5). Small intestinal mucosa was scraped using a microscope slide, then 3 g of mucosal scraping was homogenized with 300 MHT buffer (30 ml). The homogenate from the previous step was centrifuged at 2,500 g for 15 min and supernatant was saved. 3 ml of 100 mM MgCl_2_ solution (100 mM MgCl_2_, 10 mM HEPES-Tris, pH 7.5) was slowly added to the supernatant. The solution was centrifuged at 2,500 g for 15 min after stirring at 4°C for 20 min. Then the supernatant was saved and centrifuged at 50,000 g for 30 min. The pellet was resuspended MHT buffer (30 ml) and homogenized 10 times with a Dounce homogenizer, and then centrifuged at 50,000 g for 30 min. The pellet was resuspended in 30 ml of 400 MHT buffer (400 mM D-mannitol, 10 mM HEPES-Tris, pH 7.5) again, then centrifuged at 50,000 g for 30 min. BBMVs were obtained by suspending pellet in appreciate volume of 400 MHT buffer at a BBMV protein concentration of 5-10 mg/ml. Aliquots of BBMVs were stored in the tank with liquid nitrogen. Enrichment of glucose phosphatase and depletion of alkaline phosphatase were assessed as a quality control for BBMVs. BBMVs were typically 6- to 10-fold enriched.

### Measurement of glucose transport activity in BBMVs

Glucose uptake was measured in BBMVs to represent glucose transport activity from RYGB and control rats. Evaluation of glucose transport was performed by using rapid mixing filtration technique at room temperature (27). Glucose uptake was initiated by mixing 10 µl of BBMVs (-10 µg membrane protein) with 40 µl Na or choline uptake buffer (100 mM mannitol, 10 mM Tris-HEPES, 100 µM glucose containing a tracer amount of [^3^H] glucose and 125 mM NaCl or choline chloride, pH 7.4). Glucose uptake was terminated at 10 s by addition of 1 ml ice-cold wash buffer (uptake buffer without glucose) followed by rapid filtration under vacuum through a 0.45 µm membrane filter (GN-6 grid, Gelman Laboratory) and four additional washes. The filter was incubated in 5 ml of Scintisafe 30% (Cat. #: SX23-5, Fisher Scientific) for liquid scintillation counting (Beckman LS 1801, Beckman Instruments Inc, Palo Alto, CA). Transport activity was shown in picomoles of glucose per mg of protein per 10 s of uptake (Pmol GLU/md/10S). To obtain a true measure of the radioactivity level in a sample, nonspecific retention obtained from the zero-time uptake was subtracted from the total amount of radioactivity measured. Total glucose transport activity was obtained by using sodium chloride buffer. The sodium-independent glucose transport activity was obtained by using choline chloride buffer. The sodium-dependent glucose transport activity was obtained by subtracting the sodium-independent glucose transport activity from the total glucose transport activity.

### Measurement of SGLT1, GLUT2, GPR-119, FFAR2/3 protein levels

Equal amounts of protein (20 µg) were separated by SDS-PAGE on precast polyacrylamide gels (Cat. #: 25201, Pierce, Rockford, IL) and transferred to PVDF membranes (Millipore, Bedford, MA). Transfected PVDF membrane was blocked for 1 h at room temperature in TBS-T (150 mM NaCl, 10 mM Tris, 0.1% Tween 20, PH 8.0) with 3% dry milk. Then, membranes were incubated with primary antibodies (SGLT1: Cat. #: 07-1417, 1:1000, GLUT2: Cat. #: 07-1402-I, 1:1000, from Millipore Bedford, MA; FFAR2: Cat. #: 32960, 1:500; FFAR3: Cat. #: 98332, 1:500; GPR-119: Cat. #: 48195, 1:500; Tas1R1: Cat. #: 22451, 1:500, Tas1R3: Cat. #: 22458, 1:500 from Santa Cruz Biotechnology Inc, Santa Cruz, CA) in TBS-T with 3% dry milk overnight at 4°C. After three washes in TBS-T, the membranes were incubated with a HRP-conjugated secondary antibody for 1 h at room temperature. And then membranes were washed in TBS-T and proteins were visualized by Luminol Enhancer Solution (Cat. #: 46640, Thermo Scientific Rockford IL) according to the manufacturer’s instructions. Membranes were stripped by incubation with Restore Western Blot Stripping Buffer (Thermo Scientific Rockford IL) at room temperature for 10 min. β-Actin (Cat. #: sc-47778, 1:4000, Santa Cruz Biotechnology, Santa Cruz, CA) was used to verify equal protein loading. Band intensity was quantified by use of a calibrated densitometer (model GS800, Bio-Rad, Hercules, CA) using Quantity One software. Immunoblot results are reported as relative densitometry units (RDU) normalized to β-Actin.

### Measurement of proglucagon, proprotein convertase 1/3 (PC 1/3), FFAR2, FFAR3, GPR119, Tas1R1, Tas1R3 and gustducin α

The segments of small intestine were harvested and washed with ice-cold. Small intestinal mucosa was then scraped and immediately frozen in liquid nitrogen. Total RNA was isolated by the TRIzol method (Cat. #: 15596, GIBCO BRL, Life Technologies). Total RNA (2 µg) was converted to cDNA using an iScript cDNA Synthesis Kit (Cat. #: 1708891, Bio-Rad Laboratories, Inc. Hercules, CA). PCR was performed with the StepOnePlus Real-Time PCR System with StepOne software V2.0 (Applied Biosystems, Forster City, CA) according to the manufacturer’s guidelines. TaqMan Gene Expression master Mix was used to examine, Tas1R1 and Tas1R3 (Tas1R1: Rn00590759_g1, TasR3: Rn01516038_1, β-Actin: Rn00667869_m1, Applied Biosystems, Foster City, CA). iQ SYBER Green Supermix (Cat. 1708880, Bio-Rad Laboratories, Inc. Hercules, CA) was used to detect FFAR2, FFAR3, GRP119, PC 1/3. The primers utilized were 5’-TCA CCA TCT TCT GCT ATT GGC GCT-3’ (sense) and ACC AGG TGG GAC ATG TTG TAA GGT-3’ (anti-sense) for FFRA2, 5’-AGT GTA GTC TGT TGG TTC CTG GCA-3’ (sense) and TTC CAG GTA GCA GGT TCC ATT GGT-3’ (anti-sense) for FFAR3, 5’-TGG AAC CAG CAC CGT TCA GTT GG -3’ (28) and 5’-TCC ACT CCT CTC CTG TCA TTC TGG A -3’ (anti-sense) for PC 1/3, 5’-ATC CGG AAG ATG GAA GAT GCA GGA -3’ (Sense) and 5’-AGT CCG GAC AGC CTT GAA GTC ATT -3’ (anti-sense) for GPR119, 5’- CAG GGC TGC CTT CTC TTG TGA -3’ (sense) and 5’-GGC GGA GAT GAT GAC CCT TT -3’ (anti-sense) for GAPDH.

### Statistical analysis

Data were summarized using mean ± SE or median with interquartile range (IQR) depending on the distribution of the data. The number of samples in each experiment was specified in the figure legend. Differences among groups were assessed using analysis of variance (ANOVA) followed by the Tukey-Kramer post test. Statistical analysis of the data was performed using Prism 5.02 (GraphPad Software, San Diego, CA).

## Results

### Effect of RYGB on food intake and body weight

**Figure 1:**
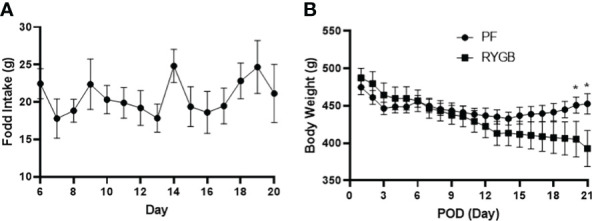
Effect of RYGB on body weight and food intake in obese Zucker rats. Body weight and food intake were monitored daily over 21-day study period after RYGB. **(A)** Daily mean food consumption (g/day) for the RYGB and PF groups. **(B)** Daily mean body weight (g) for the RYGB and PF groups. Data are mean ± SE, n=9-12 per group, *p<0.05.

Both the RYGB and PF groups were kept NPO until POD 1, started on liquids, then converted to chow on POD 3. From POD 6 to 20 food intake in the RYGB group was relatively stable at approximately 18 g per day (**Figure 1A**). PF rats were given an identical quantity of food. ZR rats fed *ad libitum* normally consume 35-40 g of chow (6). The mean body weights of the RYGB (470 g) and PF (472 g) rats were similar prior to surgery. **Figure 1B** shows the change in body weight over time. The RYGB and PF groups initially lost weight after surgery. However, on POD 3 the PF group’s body weight stabilized at 446 g, while the RYGB group continued to lose body weight. The differences in body weight between the groups became statistically significant on POD 20 (451 g vs. 405 g, p < 0.05). On POD 21 the mean weight of RYGB rats was 393g, 13% lower than the body weight of the PF rats.

### RYGB impacts peripheral and portal venous glucose and GLP-1 levels

**Figure 2:**
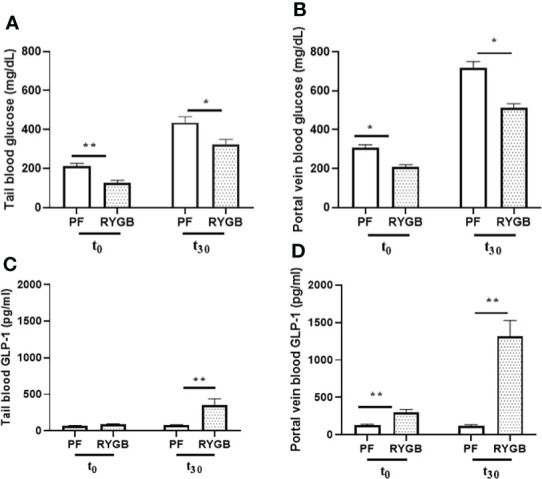
Effect of RYGB on peripheral and portal venous glucose and GLP-1 levels. On POD 21, tail and portal vein blood were collected after fasting (T_0_) and at 30 minutes (T_30_) after rats underwent gavage with 1.25g/kg 25% dextrose. Glucose levels **(A, B)** were measured by glucometer. And GLP-1 levels **(C, D)** were assayed by ELISA. Data are mean ± SE, n=9-13 per group, *p<0.05, **P<0.01.

Glucose levels in both peripheral and portal circulation have been shown to regulate appetite and systemic glucose homeostasis (29). Although several studies have examined the effects of RYGB on peripheral glucose levels, the effects of surgery on portal venous glucose levels have not previously been characterized. OGTs were performed on POD 21 in the RYGB and PF rats to assess for differences in glucose tolerance. Blood samples were collected from the peripheral and portal circulations at baseline and 30 minutes after dextrose gavage. In the peripheral circulation (**Figure 2A**) prior to OGT the fasting plasma glucose was 40% lower in the RYGB group (p<0.01 vs. PF). 30 minutes after OGT the plasma glucose levels were increased in both groups. However, the RYGB group had smaller increase in plasma glucose (194 mg/dl vs. 222 mg/dl in the PF group) and a lower absolute glucose level 322 mg/dl vs. 435 mg/dl (p<0.05 vs. PF). A similar pattern of fasting and post-prandial glucose levels was observed in plasma samples from the portal vein (**Figure 2B**). The fasting portal venous glucose level was 32% lower in the RYGB group (209 mg/dl vs. 307 mg/dl, p<0.05 vs. PF). 30 minutes after gavage, the RYGB group showed a 28% smaller increase in portal venous glucose (514 mg/dl vs. 717 mg/dl) (p <0.05 vs. PF). However, the changes in rate did not show a statistically significant difference between fasting portal venous glucose and portal venous glucose at 30 minutes after gavage.

GLP-1, one of proglucagon gene-derived peptides, is expressed in both pancreas and intestinal endocrine cells. In L-cells, the proglucagon precursor protein is processed by PC 1/3 to yield GLP-1. GLP-1 is stored in L-cell granules and released into intestinal lymphatics and the splanchnic circulation in response to luminal nutrients. Currently, available data suggest that GLP-1 release from L-cells is mediated by multiple mechanisms including: activation of multiple G-protein coupled receptors by lipids (GPR 40 and 120), lipid amides (GPR 119), short-chain fatty acid receptors (FFAR2 or GPR43, FFAR3 or GPR41) and bile acids (TGR5).

To develop a better understanding of how RYGB improves glucose homeostasis we examined the effects of RYGB on GLP-1 synthesis, as well as the regulation of intestinal proteins which mediate GLP-1 release and bioavailability. First, tail snip and portal vein blood samples from OGT were analyzed for GLP-1 protein levels using multiplexed ELISA. As shown in **Figure 2C** peripheral blood samples from the RYGB and PF rats had similarly low levels of GLP-1 (89 vs. 70 pg/ml, p > 0.05) in the fasting state. However, 30 minutes after gavage the peripheral GLP-1 levels increased almost three-fold in the RYGB rats to 354 pg/ml (p<0.01 vs. fasting). In contrast, the GLP-1 levels in the PF rats were not significantly increased 30 minutes after OGT (70 vs. 79 pg/ml). In the portal circulation (**Figure 2D**) the fasting GLP-1 level of 302 pg/ml in the RYGB group was more than double the GLP-1 level of 129 pg/ml observed in the PF rats (p<0.01). 30 minutes after dextrose gavage, the portal venous levels of GLP-1 levels increased slightly to 122 pg/ml in the PF rats. In contrast, the post-prandial portal venous levels of GLP-1 increased more than 10-fold to 1313 pg/ml (p<0.01 vs. fasting and PF post-prandial) in the RYGB group.

**Figure 3:**
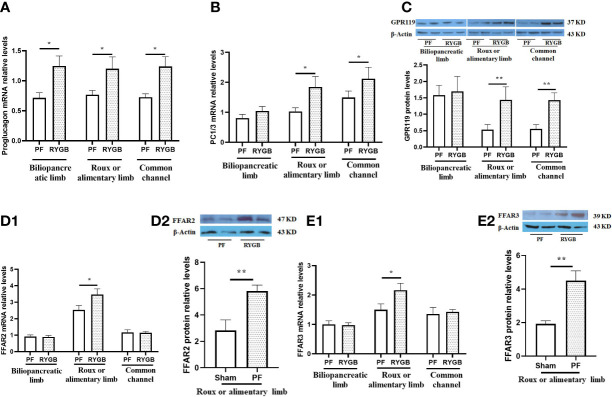
Effect of RYGB on the regulation of intestinal proglucagon and PC1/3 mRNA levels, protein levels of GPR119 and protein/mRNA of FFAR2/FFAR3. On POD 21, segments from the biliopancreatic limb (BP), Roux limb and common channel (CC) were harvested from RYGB rats along with corresponding segments from PF rats. Mucosal proglucagon **(A)**, PC1/3 **(B)**, FFAR2 **(D_1_)** and FFAR3 **(E_1_)** mRNA levels were measured by RT PCR. Mucosal GPR119 **(C)**, FFAR2 **(D_2_)** and FFAR3 **(E_2_)** protein levels were measured by Western Blot. Data are mean, ± SE, n=5-13 per group, *p<0.05, **P<0.01.

We measured the relative abundance of proglucagon mRNA in intestinal segments from the RYGB and PF rats to determine if proglucagon gene expression is increased after RYGB. As seen in **Figure 3A**, the relative abundance of proglucagon mRNA was increased in all of the mucosal samples from the RYGB group. Proglucagon mRNA levels were increased 74% in the BP limb, 56% in the Roux limb and 70% in the CC segment relative to analogous intestinal segments from the PF group. To determine if proglucagon peptide processing to GLP-1 was also increased after RYGB we examined the expression of PC 1/3 mRNA in the same samples (**Figure 3B**). There were no significant differences in PC 1/3 mRNA levels in the BP limb (1.047 vs 0.811, p > 0.05). However, the relative abundance of PC 1/3 mRNA was increased in both the Roux (1.843 vs 1.032, p< 0.05) and CC (2.121 vs 1.495, p<0.05) intestinal segments from the RYGB rats (P<0.05, vs PF).

Next, we examined the effects of RYGB on GPR119, FFAR2 and FFAR3 expression in the different intestinal segments. **Figure 3C** shows GPR119 protein levels are similar in the BP limbs. In contrast, GPR119 protein levels in RYGB rats were 170% higher in the Roux limb (0.530 vs 1.440, p<0.01) and 157% higher in the common channel (0.558 vs 1.443, p<0.01) relative to the corresponding PF intestinal segments. In addition, FFAR2 and FFAR3 mRNA (**Figures 3D_1_, E_1_**) and protein (**Figures 3D_2_, E_2_**) levels in RYGB group were significantly higher in the Roux limbs than in PF rats (mRNA P<0.01, protein p<0.05).

### Effects of RYGB on intestinal glucose transport and transporter expression

**Figure 4:**
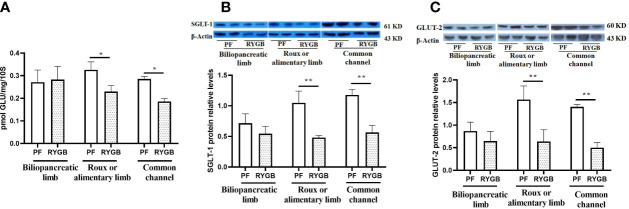
Effect of RYGB on the BBMV glucose transport and glucose transporter proteins of SGLT1 and GLUT2. On POD 21 segments from the biliopancreatic limb (BP), Roux limb and common channel (CC) were harvested from RYGB rats along with corresponding segments from PF rats. BBMVs were isolated and glucose uptake **(A)** was measured by rapid mixing uptake as described in the materials and methods. Glucose transporter proteins of SGLT1 **(B)** and GLUT2 **(C)** were examined by Western Blot. Data are mean ± SE, n=5-8 per group, *p<0.05, ** P<0.01.

The activity of glucose transport is impaired in the diabetic state (30). BBMV were isolated from intestinal mucosa samples and glucose transport was evaluated as described in Methods. As seen in **Figure 4A**, the rate of glucose transport activity was similar in the BP segments (p > 0.05). In contrast, After RYGB, glucose transport was decreased by 29% in the Roux (p<0.05 vs. PF) and 35% in the CC segments (p<0.05 vs. PF).

SGLT1 and GLUT2 play important roles in intestinal glucose sensing, transport and incretin secretion. Therefore, we assayed segments for the relative abundance of SGLT1 and GLUT2 protein levels. As shown in **Figure 4B**, the relative abundance of SGLT1 protein is decreased in Roux limb and CC segments after RYGB, falling by 54% in the Roux (p<0.01) and 52% in the CC segment (p<0.01). Relative expression of GLUT2 protein in the different intestinal segments is shown in **Figure 4C**. GLUT2 protein levels were similar in the BP segment, decreased by 53% in the Roux limb (p<0.01 vs. PF) and 64% in the CC segment (p<0.01 vs. PF).

### Effects of RYGB on intestinal Tas1R1, Tas1R3 and α-gustducin expression

**Figure 5:**
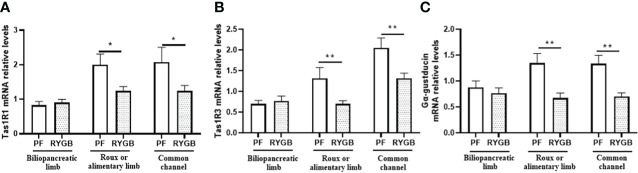
Effect of RYGB on intestinal taste receptor and α-gustducin mRNA. On POD 21, segments from the biliopancreatic limb (BP), Roux limb and common channel (CC) were harvested from RYGB rats along with corresponding segments from PF rats. Mucosal Tas1R1 **(A)**, Tas1R3 **(B)** and α-gustducin **(C)** mRNA were measured by RT-PCR. Data are mean ± SE, n=11-13 per group, *p<0.05, **p<0.01.

To determine if the effects of RYGB on intestinal glucose transport and GLP-1 secretion might be explained by altered regulation of sweet taste we examined the effects of RYGB on the expression of taste proteins Tas1R1, Tas1R3 and α-gustducin (**Figure 5**). As shown in **Figure 5A** Tas1R1 mRNA levels were similar in the BP limb (p>0.05), decreased by 38% in the Roux limb (p<0.05 vs. PF) and decreased by 40% in the CC segment (p<0.05 vs. PF). Likewise, **Figure 5B** demonstrates Tas1R3 mRNA levels to be similar in the BP limb (p>0.05), reduced by 47% in the Roux limb (p<0.01 vs PF) and reduced by 36% in the CC segment (p<0.01 vs PF). As shown in **Figure 5C** α-gustducin mRNA levels were similar in the BP limb (p>0.05), decreased by 50% in the Roux limb (p<0.01 vs PF) and reduced by 47% in the CC segment (p<0.01 vs PF).

## Discussion

The current study examines the underlying mechanisms of glucose homeostasis after RYGB using the Zucker rat model of genetic obesity. Obesity in the ZR is an autosomal recessive trait (*fa/fa*) caused by defective leptin receptors (31). The obese ZR develops progressive features of the metabolic syndrome including: insulin resistance, glucose intolerance, hyperlipidemia, and hypertension (31). Previous studies demonstrate the obese ZR is a suitable model for investigating the effects of RYGB on glucose homeostasis (26, 32). Early postoperative improvements in glycemic control, insulin sensitivity, post-prandial increases in GLP-1 and insulin secretion seen in RYGB patients are also observed after RYGB in the ZR model. Because intestinal GLP-1 production and glucose transport are a major focus of the current study, pair-feeding the control group was an important aspect of the study design. Postoperative day 21 was chosen for studies examining intestinal GLP-1 production and glucose transport based on previous results with this model (26, 27). As previously shown by our laboratory, the PF group demonstrates a slight reduction in body weight compared to the RYGB group, but insulin sensitivity and GLP-1 secretion are not affected in the PF group during the study period (26).

Consistent with previous results, RYGB improves both fasting and post-prandial peripheral glucose levels compared to the PF group. We examined both portal venous glucose and GLP-1 levels because of the potentially important roles they play in regulating systemic glucose homeostasis (33), rapid insulin secretion (34), glucose uptake (35) and hepatic gluconeogenesis (36). After RYGB surgery both the basal and 30-minute post-prandial portal venous glucose levels were decreased relative to those in the PF controls. The reductions in portal venous glucose after RYGB could be due to decreased peripheral glucose levels, reductions in enteric glucose absorption and transport, down regulation of intestinal gluconeogenesis, increased hepatic glucose uptake and/or metabolism or some combination of these factors. Intestinal gluconeogenesis is a key factor in regulating insulin sensitivity and food intake is independent of body weight and GLP-1 action after RYGB. However, reductions in intestinal phosphoenolpyruvate carboxykinase (PEPCK-C) and glucose-6-phosphatase (G6Pase) after RYGB suggest intestinal gluconeogenesis is down regulated (37). Therefore, we examined the effects of RYGB on GLP-1 synthesis and secretion, as well as intestinal glucose transport in the current study.

Several lines of evidence implicate intestinal production of GLP-1 as a major factor in the early postoperative glucose hemostasis observed after RYGB surgery (38). GLP-1 is an incretin hormone produced by enteroendocrine L cells located in the epithelial layer of the jejunum, ileum and colon. Intra-luminal nutrients, including glucose and lipids trigger GLP-1 release into the intestinal interstitial space and lymphatics where it travels from the portal vein, through the liver into the systemic circulation. GLP-1 binds beta cell GLP-1 receptors resulting in enhanced glucose-stimulated insulin secretion. Salehi et al. used exendin- (9–39) in post-RYGB patients to investigate the role of systemic GLP-1 in regulating postprandial insulin secretion in the subjects with Gastric bypass (39, 40). Their results suggest GLP-1 stimulated insulin secretion contributes significantly to improved glucose homeostasis after RYGB surgery. Despite this finding, the role of GLP-1 in regulating glucose homeostasis appears to be significantly more complex than its endocrine role in stimulating pancreatic insulin secretion.

The reduction in portal venous glucose and increase in portal GLP-1 support the hypothesis that changes in intestinal metabolism after RYGB play an important role in glucose homeostasis. This is among the first studies to characterize the effects of RYGB on portal venous glucose and GLP-1. The gut acts as an entero-endocrine organ through the secretion of incretins including GLP-1, secreted by L cells in the distal small bowel. GLP-1 binds specific receptors on pancreatic β-cells to increase islet cell mass and stimulate insulin secretion (41–43). Extrapancreatic effects of GLP-1 include the stimulation of glucose metabolism in liver and muscle. Furthermore, portal venous GLP-1 has been shown to have insulin-independent effects on circulating glucose levels (44). Both GIP and GLP-1 signaling pathways are impaired in T2DM.

We have previously shown that while fasting peripheral GLP-1 levels are similar among RYGB and control rats, only the RYGB rats mount a response to OGT with a spike GLP-1 levels. In addition to confirming our previous results, we made the novel finding that in contrast to the peripheral blood, fasting portal venous GLP-1 levels are significantly higher in RYGB rats versus controls. While control rats again fail to respond to OGT, the increase in GLP-1 protein levels among RYGB rats is much higher in the portal venous blood than was seen in the periphery. Proglucagon mRNA levels were significantly elevated in all three intestinal segments after RYGB, suggesting a global upregulation of intestinal GLP-1 synthesis. Whereas the levels of PC1/3 mRNA were only increased in the Roux and common channel segments. The PC1/3 enzyme converts proglucagon into GLP-1. The increase of both proglucagon and PC1/3 mRNA levels after RYGB supports increased GLP-1 synthesis and secretion as the mechanism for increased circulating GLP-1 after RYGB versus reduced GLP-1 activity or degradation by DPP-4. We also measured the levels of intestinal GPR119, a G protein-coupled receptor expressed in enteroendocrine cells, which senses fats/lipids resulting in GLP-1 secretion (45). The increased the level of GPR119 observed in Roux and common channel intestinal segments supports this concept. Another possibility is that nutrient sensing free fatty acid receptors (FFARs) like FFAR2 and FFAR3 on enteroendocrine cells are upregulated and contribute to increased GLP-1 secretion (46–50). The increased the levels of FFAR2/3 mRNA and protein found in Roux intestinal segment suggest they contribute to the increases in GLP-1 observed after RYGB.

Finally, we looked at changes in nutrient transport following RYGB. By isolating brush border membrane vesicles, we were able to measure glucose transport across the intestinal lumen and found that while there was no change in the biliopancreatic limb, RYGB rats showed significant reductions in glucose transport in the Roux and common channel limbs compared to controls. The current model of intestinal glucose transport posits active brush border transport *via* SGLT1 and facilitated basolateral glucose transport following apical translocation of GLUT2 (17). The reductions in SGLT1 and GLUT2 protein levels observed in the Roux and common channel segments after RYGB are consistent with the decrease in intestinal glucose transport observed after RYGB and represent a potential mechanism. Consistent our findings, Stearns et al. (51) showed that glucose transport is reduced by up to 68% in the Roux limb compared to sham jejunum. Work by Kellet (52) and others posits the SGLT1 pathway, though easily saturated, provides the initial signal for apical translocation of the high capacity GLUT2 transporter.

Mace and others (18, 23) propose that a second signal is required to complete insertion of GLUT2 into the membrane and that this is derived from taste receptors including Tas1R2, Tas1R3 and gustducin. In addition, work from Margolskee (24, 53) supports Tas1R2, Tas1R3 and gustducin mediated sweet tastant sensing in L cells as a mechanism for controlling GLP-1 secretion and SGLT1 expression (54, 55). Accordingly, we examined Tas1R1, Tas1R3 and gustducin mRNA levels and found that they were significantly decreased in the Roux and common channel limbs, but not in the biliopancreatic limb of RYGB rats compared with controls.

Taken together these data suggest that RYGB alters the expression of taste receptors in the distal segments of intestine by exposing them to higher than normal nutrient flow. This increased signaling through GRP119, FFAR2/3 and their downstream effects through GLP-1 and SGLT1/GLUT2 mediated by sweet taste receptors represent a potential mechanism for improved glycemic control in diabetes following RYGB gastric bypass.

Although our results provide insights into potential mechanisms by which RYGB improve glucose homeostasis, our study has some limitations. One is that findings in experimental models of obesity and T2DM, like the ZRs, do not always apply to the human condition. The use of only two data points for portal and systemic glucose and GLP-1 measurement is another limitation. The two time points were based on previous studies and the inherent risks of obtaining multiple portal venous blood samples. Another limitation is that measurement of glucose transport *in vitro* using rapid mixing filtration techniques, may not completely recapitulate *in vivo* glucose uptake under various physiological conditions. Although we quantified SGLT1 and GLUT-2 levels in intestinal segments, we did not confirm their cellular localization using immunohistochemistry in our experiments, instead relying on previous studies characterizing their cellular localization (27). We also did not fully characterize the changes in potential regulatory molecules (e.g. GPR119, FFARs and taste receptors) to prove cause and effect for observed changes in glucose uptake or GLP-1 secretion after RYGB.

In summary, our results identify multiple changes in intestinal metabolism after RYGB that contribute to decrease blood glucose and increase post-prandial GLP-1. The increase in GLP-1 appears to be the result of increased proglucagon synthesis and processing, as well as upregulation of multiple nutrient-sensing receptors (e.g. GPR119, FFAR2/FFAR3) that may stimulate GLP-1 release from enteroendocrine cells although the causal relationship between them needs to be further defined. Post-RYGB, reductions in intestinal glucose transport appear to be regulated by decreased expression of SGLT1 and GLUT2. Although associated reductions in taste receptors (Tas1R2, Tas1R3 and gustducin) represent a potential mechanism, additional studies are needed to prove cause and effect.

## Data availability statement

The original contributions presented in the study are included in the article/supplementary material. Further inquiries can be directed to the corresponding author.

## Ethics statement

The animal study was reviewed and approved by Institutional Animal Care and Use Committee at the Pennsylvania State University, College of Medicine.

## Author contributions

RC contributed to study concept and experimental design. QM, DC, MS, and TA helped with performing surgery, specimen collection and analysis, as well as interpretation of data. QM prepared the figures. QM and RC contributed to the writing and editing of this manuscript. All authors provided contributions to this study and approved the submitted version.

## Acknowledgments

QM and RNC acknowledge the support of NIH R01 DK122332 for this work.

## Conflict of interest

The authors declare that the research was conducted in the absence of any commercial or financial relationships that could be construed as a potential conflict of interest.

## Publisher’s note

All claims expressed in this article are solely those of the authors and do not necessarily represent those of their affiliated organizations, or those of the publisher, the editors and the reviewers. Any product that may be evaluated in this article, or claim that may be made by its manufacturer, is not guaranteed or endorsed by the publisher.
